# Socioeconomic position and body composition in childhood in high- and middle-income countries: a systematic review and narrative synthesis

**DOI:** 10.1038/s41366-021-00899-y

**Published:** 2021-07-27

**Authors:** Charis Bridger Staatz, Yvonne Kelly, Rebecca E. Lacey, Joanna M. Blodgett, Anitha George, Megan Arnot, Emma Walker, Rebecca Hardy

**Affiliations:** 1 Social Research Institute, Institute of Education, University College London, London, UK; 2 Department of Epidemiology and Public Health, University College London, London, UK; 3 Institute of Sport Exercise and Health, Division of Surgery and Interventional Science, Faculty of Medical Sciences, University College London, London, UK; 4 Department of Anthropology, University College London, London, UK

**Keywords:** Risk factors, Epidemiology

## Abstract

**Background:**

The relation between socioeconomic position (SEP) and obesity measured by body mass index (BMI), a measure of weight for height, has been extensively reviewed in children, showing consistent associations between disadvantaged SEP and higher BMI in high-income countries (HICs) and lower BMI in middle-income countries (MICs). Fat mass (FM), a more accurate measure of adiposity, and fat-free mass (FFM) are not captured by BMI, but have been shown to track from childhood to adulthood, and be important for cardiovascular health and functional outcomes in later life. It is not clear whether body composition is associated with SEP. We systematically reviewed the association between SEP and body composition in childhood.

**Methods:**

A systematic review was carried out following PRISMA guidelines. The protocol was pre-registered with PROSPERO (CRD42019119937). Original studies in the English language, which examined the association between SEP and body composition in childhood, were included. An electronic search of three databases was conducted. Two independent reviewers carried out screening, data extraction and quality assessment. Due to heterogeneity in results, a narrative synthesis was conducted. Heterogeneity in findings according to SEP, sex, body composition measure and country income level was investigated.

**Results:**

50 papers were included, the majority from HICs. No papers were from low-income countries. Disadvantage in childhood was associated with greater FM and lower FFM in HICs, but with lower FM and lower FFM in MICs. When measures of FFM indexed to height were used there was no evidence of associations with SEP. In HICs, more studies reported associations between disadvantaged SEP and higher FM among girls comparative to boys.

**Conclusions:**

Inequalities in FM are evident in HICs and, in the opposite direction, in MICs and follow similar trends to inequalities for BMI. Inequalities in height are likely important in understanding inequalities in FFM.

Childhood obesity is a globally recognised public health challenge and is a major determinant of obesity in adulthood [1]. Previous systematic reviews investigating the link between socioeconomic position (SEP) and obesity measured through body mass index (BMI) in childhood have predominantly found disadvantaged SEP to be associated with higher levels of obesity in high-income countries [2, 3], especially when SEP was measured by parental education [2]. In low- and middle-income countries, disadvantaged SEP is associated with lower levels of obesity [4]. Compared with studies in adults where sex differences have been observed, with women typically demonstrating greater evidence of inequalities, little evidence of stronger associations in girls compared to boys in high-income countries has been found [2].

The majority of evidence on inequalities in overweight and obesity in children comes from studies using BMI, a measure of weight for height which does not distinguish fat mass (FM) from fat-free mass (FFM) and therefore may under or overestimate adiposity. Measures of body composition can provide information about the location of FM and estimates of the proportion of FM to FFM. FFM includes bone mass and lean mass (LM) and is most frequently measured by bioelectrical impedance analysis (BIA). LM is a measure that excludes bone mass and is most frequently measured through dual x-ray absorptiometry (DXA) [5]. In adults, a higher proportion of fat-to-lean mass is associated with a higher risk of cardiovascular disease [6]. Both total and proportion of fat mass have been associated with cardiovascular and metabolic disease, with higher central adiposity and android-to-gynoid fat mass ratio implicated in increased risk [7–10]. In addition, LM plays a role in development of insulin sensitivity, with muscle tissue being a site of glucose uptake, therefore having the potential to reduce and delay the onset of metabolic disorders [11, 12].

Studies using serial data in children have shown secular changes in body composition, with an increasing trend for FM index (FMI) from 1960 to 1999 in the US [13]. In the UK, decline in muscle fitness, as measured by strength, power and strength-endurance, has been observed among children, when adjusted for height and weight, between 1998 and 2014 [14]. It is possible that such secular changes in body composition are accompanied by increases in socioeconomic inequality in body composition, as has been observed for BMI [15], where increases in inequalities are particularly evident across childhood [16]. Additionally, secular changes in muscle and fat acquisition in childhood may subsequently result in detrimental secular changes in adult body composition. As people age, BMI increases are more likely to reflect fat acquisition than muscle [17] and tracking of body composition from childhood to adulthood has been demonstrated [18, 19].

We therefore carried out a systematic review to assess the association between SEP and measures of body composition (in particular FM, FFM, and the location of FM) in children (up to and including 18 years) from general population samples. Additional aims were to assess secular changes in socioeconomic inequalities in body composition and explore heterogeneity by sex, SEP measure, body composition measure and income level of country of study.

## Methods

The protocol for this review, which is the second part of a larger systematic review investigating socioeconomic inequalities in adults [20], was registered with the PROSPERO database (CRD42019119937). The review has been carried out according to Preferred Reporting Items for Systematic Reviews and Meta-Analyses (PRISMA) checklist (Supplementary File 1). Further details of the methods can be found in the published protocol [21].

### Eligibility

Peer reviewed papers written in the English language were included in this review if they reported an association between SEP and body composition in children (under 18 years of age) using data from an observational study including a sample from the general population. Associations between any recognised indicator of SEP (e.g., income, education, overcrowding, area-level deprivation) and a measure of body composition, measured at the same, or later, time point to SEP were included. As studies in this review are in children, measures of SEP, such as occupation and education, relate to parents, other markers such as overcrowding reflect the home in which the child lives, and some markers of education are based on the type of school attended. Body composition (i.e., measured using BIA or DXA) was defined as any measurement related to total FM and FFM, location of FM and FFM or any proportion or ratio of measures of FM and FFM.

### Search strategy

CBS conducted an electronic search of three databases (MEDLINE and Embase Classic + Embase using OvidSP as the interface, SPORTDiscus using EBSCO as the interface) from the earliest entry up until the 30th of January 2019. The search terms used are shown in Table 1 and include adult as well as childhood samples. The results of the search were de-duplicated and stored in the reference manager, Endnote. This database was exported to Rayyan Qatar Computing Research Institute (QCRI) [22] to conduct screening. CBS, AG, and JB conducted title and abstract screening for eligibility, and subsequent full text screening of eligible papers for inclusion in the review. Additionally, the reference list of eligible full texts were screened and searches of publications from key studies with relevant data were used to identify further papers.

**Table 1:** Search terms.

Search terms	
Database	MeSH Terms
Medline	Body Composition – exp Body Composition/; Adipose Tissue/; exp Body Fat Distribution/; Obesity/or obesity, abdominal/.
	Body Composition Measures - Electric Impedance/; Magnetic Resonance Imaging/; Tomography, X-Ray Computed/; Densitometry/; Whole-Body Counting/; Plethysmography/.
	Socioeconomic Position - socioeconomic factors/ or poverty/ or poverty areas/ or social class/; Educational status/ or income/ or occupations/ or social conditions/.
Embase + Embase Classic	Body Composition - Body composition/ or body distribution/ or body fat/ or body fat distribution/; Obesity/; lean body weight/; Fat mass/.
	Body Composition Measures - Impedance/; nuclear magnetic resonance imaging/; computer assisted tomography/; densitometry/; whole-body counting/; Total body water/; plethysmography/.
	Socioeconomic Position - socioeconomics/ or educational status/ or income group/ or poverty/; income/ or occupation/ or household income/; social status/ or social background/ or social class/; education/;
SPORTDiscuss	Body Composition - ((DE “BODY composition” OR DE “HUMAN body composition”) OR (DE “OBESITY”)) OR (DE “ADIPOSE tissues”)
	Body Composition Measures - ((((DE “BIOELECTRIC impedance”) OR (DE “COMPUTED tomography”)) OR (DE “MAGNETIC resonance imaging”)) OR (DE “BONE densitometry”)) OR (DE “PLETHYSMOGRAPHY”)
	Socioeconomic Position - ((DE “EDUCATION”) OR (DE “EDUCATIONAL attainment”)) OR (DE “HEALTH & income”)
Free Text Search Terms	
Body composition	1. Body Composition MeSH Terms
	2. (Body adj3 (composition or distribution))
	3. ((fat or adipos*) adj3 (composition or distribution or mass or index or kg or total))
	4. ((muscl* or lean) adj3 (composition or distribution or mass or index or kg or total))
	5. ((fat-free) adj3 (mass or kg or total))
	6. ((android or gynoid or visceral or appendicular or abdominal or intra-abdominal) adj3 (fat or lean or muscle or mass or adipos*))
	7. 1 OR 2 OR 3 OR 4 OR 5 OR 6
Body composition Measures	
	8. Body Composition Measures MeSH Terms
	9. ((impedance) adj3 (bioelectrical or foot-to-foot or hand-to-foot or analy?is))
	10. (bioimpedance or body fat analy?er or body composition analy?er or tanita)
	11. (dual x-ray absorptiometry or DEXA or DXA or dual-energy X-ray absorptiometry)
	12. (magnetic resonance imaging or MRI)
	13. (Computed tomography or CT or CAT scan)
	14. (densitometry)
	15. ((neuron activation or total body counting or whole-body counting))
	16. (total body water)
	17. (air-displacement plethysmography)
	18. 8 OR 9 OR 10 OR 11 OR 12 OR 13 OR 14 OR 15 OR 16 OR 17
	19. 7 AND 18
Socioeconomic position	20. Socioeconomic Position MeSH terms
	21. (social class or social status or social position or socio-economic or socioeconomic or social circumstance*)
	22. (sociodemo*)
	23. Occupation*
	24. Educat*
	25. (income* or manual or class)
	26. (depriv* or poverty or overcrowding)
	27. 20 OR 21 Or 22 OR 23 OR 24 OR 25 OR 26
	28. 19 AND 27
	29. Limit to English Language (and Human in OvidSP)

MeSH terms are main heading descriptor terms available in each database and are determined by the indexing method adopted by each database. Free text search terms were entered into all databases, along with the results of the database specific MeSH terms.

### Extraction and quality assessment

Relevant information was double extracted using a data extraction form by CBS, AG, JB, MA, and EW. Data extracted included citation details (author, title, publication year, publication type), study details (cohort or sample description, study design, country, participant numbers), participant details (birth year or age of participants, sex of participants), exposure and outcome details (type of SEP and body composition variables presented, age at which variables were recorded, how the variables were ascertained and measured) and statistical methods and information on adjustment for potential confounders and mediators. All available statistics relating to the association under study were extracted, along with statements of direction in text where statistics were not presented.

Assessment of study quality was carried out by CBS, AG, JB, MA, and EW, using an amended version of the Newcastle-Ottawa Quality Assessment scale [23]. Quality assessment was not used to exclude papers from the review, but to inform on the variability of quality across the papers and potential bias arising. The quality assessment form was amended after the protocol was published to account for the variability in statistical reporting and the large number of cross-sectional studies identified (questions 3bi, 3bii and 4 - Supplementary File 2). Google Forms was used to aid extraction and WebPlotDigitizer [24] was used to extract data only presented in graphs.

Two reviewers (CBS and either AG, JB, MA or EW) worked independently to complete screening, quality assessment and data extraction. Any disagreements were resolved through discussion.

### Synthesis

A meta-analysis was not possible due to the considerable variability in analytic methods used and presentation of results. As such, it was not possible to assess the degree of publication bias across studies through use of a funnel plot. Instead, a narrative synthesis was conducted, guided by the Economic and Social Research Council Methods Programme guidelines [25], with a focus on identifying and exploring sources of heterogeneity. The current review reports results only for associations in childhood due to the large number of papers included. The results for adulthood are reported elsewhere [20].

Multiple relevant associations were frequently presented in a single paper. The individual association, as opposed to the paper, were thus considered the unit of analysis, similar to methods adopted by McLaren [26], and Ball and Crawford [27]. This will have resulted in greater contribution of results from a single paper where multiple associations were reported.

Each association reported was categorised as either a positive association (those reporting greater socioeconomic advantage associated with higher body composition measure), negative association (those reporting greater socioeconomic advantage associated with lower body composition measures), non-linear association or no association. We removed the non-linear group from the summary tables, similar to the approach of McLaren [26], as only one association fell into this category. Associations were assigned to groups based on the effect estimates and 95% confidence intervals. Where estimates were not reported, assignment was based on trends identified in descriptive data or statements of direction reported in text alongside *P* values. Use of *P* values on their own only occurred if they indicated a non-significant relationship in absence of information on the direction of association.

As outlined in the protocol, heterogeneity in associations was explored according to body composition measure (FM, FFM, ratio and distribution), SEP measure and sex. Results from analyses using boys and girls combined were the primary results selected for summary. Where results were only presented for girls and boys separately, both associations were included in the summary results. It was not possible to investigate differences in body composition by birth year as outlined in the protocol, due to lack of information provided on birth year across studies. On extraction, it became clear that country income level should also be considered a source of heterogeneity. Studies were thus categorised into those in high-income countries (HIC), upper middle and lower middle-income countries, according to the World Bank classification in 2019 [28]. Those papers from “upper middle” and “lower middle” income countries will all be referred to as “middle-income countries” (MIC). On the suggestion of a reviewer, we also investigated impact of paper quality on the findings.

## Results

**Fig. 1:**
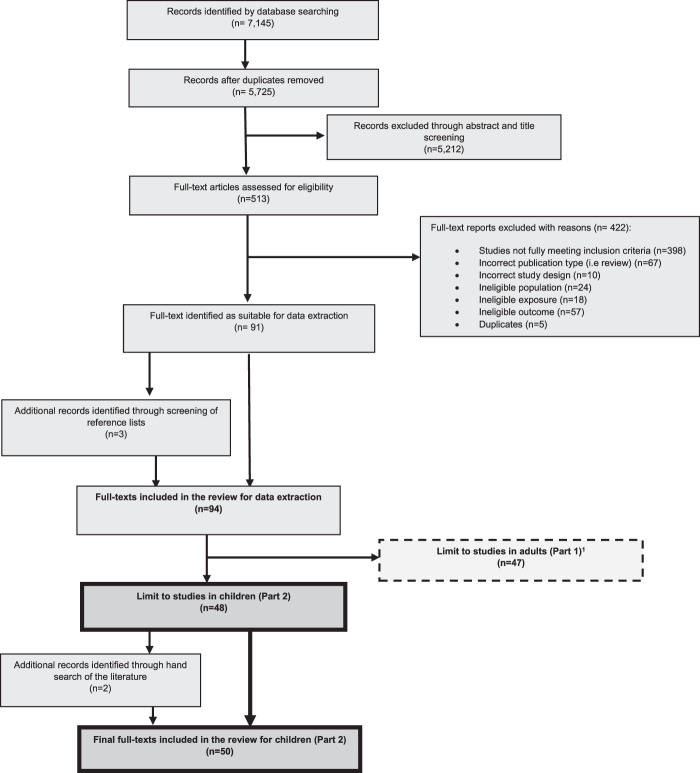
Study selection process outlined with PRISMA flow chart. Because reasons for exclusion are not mutually exclusive, numbers given for reasons for exclusion during full text-screening stage equal more than the total excluded at this stage (*n* = 422). Number of studies identified in adults and children is greater than total full texts included in the review, as one paper covered childhood and adulthood and was used in both reviews. ^1^ Studies in adults are reported on elsewhere.

In total, 7145 papers were identified from the database searches for studies in both children and adults, with 5725 once duplicates were removed. Title and abstract screening resulted in 513 papers, with 92 papers remaining following full text screening. Searching the reference lists for additional papers returned three, bringing the total included papers to 95. Of those, 48 investigated associations between SEP and childhood body composition. A search of papers from key studies resulted in two further papers in children being identified, bringing the total number of included papers to 50 [18, 29–77]. The selection process, as outlined in the PRISMA flow chart, is shown in Fig. 1. Descriptive results for the included papers are shown in Table 2. The majority of papers were rated as medium quality in the adapted Newcastle-Ottawa assessment and eight studies were rated as high quality (≥7*) and ten as low quality (≤3*). Those rated as high quality all presented full statistical results, including effects estimates and confidence intervals, whilst those rated as low quality typically had statistical reporting deemed inappropriate or incomplete. Only one paper presented *P* values alone to report a non-significant result without provision of effect estimates, descriptive data or statement of direction in text.

**Table 2:** Descriptive characteristics of included studies.

First Author	Year published	Country	Data set used (if named)/ Description of sample	*N*	SEP measures	Body composition measures	Technique	Age	Quality score
Apouey [29]	2016	UK	MCS	14,314	Family Income	FM	BIA	6–8 and 10–12	6^*^
Azcorra [30]	2016	Mexico	Mother–child dyads taken from two cross-sectional studies from Merida, Mexico. Recruited through public and private schools with diverse socioeconomic backgrounds.	197	SEI (composite score using mother’s education, father’s occupation and household crowding)	FMI	BIA	6–10 (mean: 8.53)	6^*^
Baird [31]	2016	UK	Southampton Women’s Survey	587	Mothers Education; Parental Education	FMI; FFMI	BIA	Mean: 4.1 (0.1) SEP measured before age of 3	7^*^
Boot [32]	1997	Netherlands	Caucasian children and adolescents from three primary and one secondary school in Rotterdam.	403	Parental Occupation; Fathers Education; Mothers Education	FM; %FM; LM	Both BIA and DXA	4–20	3^*^
Brown [33]	2011	USA	Children in either Kindergarten or third grade in eight elementary schools in the Hilo, Hawaii were invited to participate, with oversampling of native Hawaiian children.	125	Household Income; Mothers Education; Fathers Education	%FM; FM	BIA and plethysmograph (‘Bod Pod’)	Two age groups: Kindergarten mean age 5.6, Third grade mean age 8.7	4^*^
Burdette [34]	2006	USA	Preschool aged children part of a prospective cohort, born full term or after, without chronic health problems affecting growth and development, and with parents either both black or both white.	313	Maternal Education; Household Income	FM	DXA	4.8–5.2 (mean 5.0) SEP measured age 3.3 (0.3)	4^*^
Cardel [35]	2012	USA	Children self-identifying as African American, European American, or Hispanic American from Birmingham, Alabama area.	267	SEP (Hollingshead 4-factor index of social class)	Trunk FM; TAAT	DXA	7–12 (mean: 9.4–9.7)	2^*^
Carter [36]	2011	New Zealand	FLAME	244	Mothers Education; Income	FMI; FFMI	Both BIA and DXA	Mean 7 SEP measures age 3	8^*^
Castro [37]	2017	Brazil	Women randomly selected from large maternity hospital in São Paulo city, Brazil.	210	SES (measured by maternal education and housing conditions)	%FM	Air-displacement plethysmography	39.42 (weeks) Gestational age	4^*^
Cheng [18]	2009	Finland	Girls contacted through class teachers in 61 schools in Jyväskylä and its surroundings in Central Finland.	236	Parents Education	FM; LM	DXA	Baseline age 9–13, followed up at multiple points over 7.5 years	6^*^
Chomtho [38]	2008	UK	Healthy children born term from Greater London and Cambridgeshire, recruited through advertisements in schools and sport clubs, the intranet, local newspapers, and word of mouth.	391	Social Class (using the standard occupational classification)	FMI; FFMI	4C Model (combination of Air-displacement plethysmography, Deuterium dilution, DXA)	4–20 (mean: 11.7)	2^*^
Collings [39]	2015	UK	The ROOTS prospective cohort study	728	Area Level SES (based on post code)	FMI	BIA	Baseline mean 15, follow up at 17.5	4^*^
Datta Banik [40]	2014a	Mexico	Purposive non-probability sample of adolescents from public and private schools in Merida, Yucatan.	321	SES (based on school type, parents’ education, fathers occupation, monthly household food expenditure, crowding index); Mothers Occupation	FM%; DLM	BIA	15–17 (mean: 16.41)	4^*^
Datta Banik^a^ [41]	2011	Mexico	Cross sectional study of 13–14 boys from schools in Merida, Mexico.	74	SES (based on school type, parents’ education, fathers’ occupation and per capita monthly household food expenditure)	DLM; %FFM; %FM; FM	BIA	13–14	2^*^
Datta Banik [42]	2014b	Mexico	Purposive, non-probability sample of adolescents selected from public and private schools in Merida, Yucatan.	270	School Type; Mothers Education; SES (based on mothers’ education, fathers occupation, type of school (e.g. private), type of medical care (e.g. private))	%FM; %FFM; DLM	BIA	12–16 (mean boys: 14.01; mean girls: 13.90)	3^*^
De Vriendt [43]	2011	Multiple^c^	HELENA-CSS	1121	Parents Education	%FM	BIA	12.5–17.5 (mean boys: 14.7; mean girls: 14.8)	6^*^
Dowda [44]	2017	USA	TRACK	658	Parental Education	FMI; %FM	BIA	Baseline mean 10.6, followed for two school years	6*
Duncan [45]	2008	New Zealand	Children randomly selected from 27 primary schools in Auckland, /new Zealand.	1229	School level SES (estimated using Ministry of Education decile classification system)	%FM^b^	BIA	5–11 (mean 8.4)	6*
Ebenegger [46]	2011	Switzerland	Randomly selected kindergarten children from 40 classes with high migrant prevalence in two Swiss Cantons.	542	Parents Education; Mothers Education; Fathers Education	FM; %FM	BIA	Mean: 5.1	4*
Ekelund [47]	2005	Sweden	SWEDES	445	Maternal Education	FM; %FM;	Air-displacement plethysmograph	Mean boys: 16.9 Mean girls: 16.8	6*
Ekelund [48]	2006	Sweden	SWEDES	248	Mothers Occupation	%FM; FM; FFM	Air-displacement plethysmograph	Mean boys: 16.9 Mean girls: 16.8	8*
Gracia-Marco [49]	2012	Spain	HELENA	322	Affluence Scale; Mothers education; Fathers Education; Mothers Occupation; Fathers Occupation	LM	DXA	12.5–17.5 (mean 14.8)	3*
Griffiths [50]	2013	South Africa	Bt20	346	Maternal Education; Home Ownership; Index of School Environment; Neighbourhood Economic Index	FM, LM	DXA	Mean: 16 SEP measures in infancy and age 16	7*
Griffiths [51]	2008	South Africa	Bt20	281	SES index (created using PCA of multiple measures)	FMI; LMI	DXA	9–10 (mean 9.72) SEP measures at birth and 9–10	7*
Hou [52]	2014	Hong Kong	Hong Kong’s ‘Children of 1997’birth cohort	502	Parental Education	%ASM; %FM	DXA	15.3 SEP assumed to be measured at birth (exact age not given)	8*
Howe [53]	2010	UK	ALSPAC	7772	SII for Mother’s Education	FM	DXA	Mean 9.9 SEP collected at 32 weeks gestation	6*
Howe [54]	2013	UK	ALSPAC	6702	Mothers Education	FM	DXA	Mean 9	6*
Johnson [55]	2008	UK	ALSPAC	509	Mothers Education	FMI	DXA	Mean 9 SEP collected at 32 weeks gestation	4*
Khadilkar [56]	2012	India	Randomly selected girls from higher and lower socio-economic stratum schools and colleges in Pune, India.	390	SES (kuppuswamy socioeconomic scale)	LM; FM	DXA	8–17 (mean 12.6)	4*
Lagoa^a^ [57]	2014	Portugal	Children from 6 schools in the Porto district, Portugal.	566	Fathers SES; Mothers SES	%FM	BIA	Children	3*
Lantz [58]	2008	Sweden	Random selection of adolescents from population register from industrial town Trollhättan, Sweden.	203 and 149 at respective ages	Fathers Education	FM; LM; %FM; %LM	DXA	Two age groups: 15 and 17	6*
Magalhaes [59]	2012	Brazil	Children aged 4–7 from a retrospective cohort who were monitored for the first months of life by a support program to breastfeeding (PROLAC) in the city of Vicosa, southeast Brazil.	185	Mothers Education; Income per capita	%FM^b^; %Android Fat;	DXA	4–7 (mean 6)	5*
Matsudo [60]	2016	Brazil	ISCOLE	485	School Type; Income; Maternal Education; Fathers Education	%FM^b^	BIA	9–11	6*
McCarthy^a^ [61]	2015	UK	Caucasian children from inner city London and from more affluent surrounding counties.	2297	School Level SEP (based on percentage of children eligible for free school meals)	FFM; ASM; %FFM; ASM as % of Body Weight; Muscle:Fat Ratio; ASM as % of FFM	BIA	5–14	4*
Molina-Garcia [62]	2017	Spain	IPEN	265	Area -level SES (measured by educational level of census blocks)	%FM	BIA	14–18 (mean 16.4)	5*
Moon [63]	2018	Korea	KHANES	1233	Household Income	%ASM	DXA	12–18 (mean from 13.68 to 15.63)	3*
Ness [64]	2005	UK	ALSPAC	5917	Social Class (using the1991 UK Office of Population Censuses and Surveys classification based on occupation)	FM; LM; Trunk FM	DXA	9.9, SEP measured 32 weeks gestation	6*
Nguyen [65]	2012	USA	NHANES	5436	PIR	FM; %FM;	DXA	8–19	7*
Nguyen^a^ [66]	2011	USA	NHANES	7479	PIR	%FM^b^; FMI	DXA	8–19	4*
Plachta-Danielzik^a^ [67]	2015	Germany	Kiel Obesity Prevention Study	5352	Parental Education	FM	BIA	5–16	2*
Samani-Radia [68]	2011	UK	Subjects taken from two previous data sets, the first from children in East London and the second from children living in Hertfordshire, Cambridgeshire and West London.	2298	School Level SEP (based on school location and percentage of children eligible for free school meals)	%FM^b^; z-%FM	BIA	5–14 (mean 8.86)	5*
Santos [69]	2014	Brazil	Pelotas Birth Cohort	3350	SES; Maternal Education	FM; %FM; FMI; FFM; %FFM	Air-displacement plethysmography	Mean: 6.8	5*
Schaefer [70]	2009	USA	ACT	144	Free or reduced meal program (FRMP)	%FM	DXA	Mean: 11.6	3*
Shakir [71]	2018	AUS	Data from large multi-centre case-control study of obese and healthy weight adolescents from three Australian states.	234	Household Income	%FM	DXA	10–13 (mean: 11.9)	4*
Ulbricht [72]	2018	Brazil	Adolescents meeting inclusion criteria (parents authorised, not taking medicine containing calcium, haven’t undergone radiography/computed tomography a week prior, and were not suspecting pregnancy) from the city of Curitiba-PR, Brazil, and 29 other municipalities.	675	SEP (based on purchasing power of families)	%FM^b^	DXA	11–18 (mean: 14.7)	4*
Van den Berg [73]	2012	Netherlands	ABCD	1965	Mothers Education	FMI; LMI; %FM;	BIA	5–6 (mean: 5.7)	7*
Veena [74]	2014	India	Mysore Parthenon Birth Cohort Study	540	Standard of living Index; Mothers Education; Fathers Education; Occupation; Income (head of the family)	%FM	BIA	9–10 (mean: 9.7)	6*
Willig [75]	2011	USA	Children from ongoing cross-sectional study, whose parents classified them through self-report as either African American, European American, or Hispanic American.	254	SEP (Hollingshead 4-factor index of social class)	FMI; FFMI Trunk FMI	DXA	7–12 (means range from 9.4–9.6)	5*
Wohlfahrt-Veje [76]	2014	Denmark	Danish Population-Based Mother–Child Cohort	950	SEP (based on parental education and occupation)	%FM	DXA	6–15	5*
Zanini [77]	2014	Brazil	Pelotas Birth Cohort	3373	SEI (Constructed through PCA based on consumer goods and education of head of family); Mothers Education	FM;LM; %FM; %LM; FMI; LMI	DXA	6–7 (mean 6.7) SEP measures from perinatal study	6*

Where papers have reported either body fat or fat mass, the variable is listed as just fat mass.

*SEI* Socioeconomic Index, *SIMD* Scottish Index of Multiple Deprivation, *SEP* Socioeconomic Position, *SES* Socioeconomic Status, *SII* Slope of Inequality Index, *PIR* Poverty Income Ratio, *FRMP* Free or Reduced Meal Program, *PCA* Principle Component Analysis, *FM* Fat Mass, *FFM* Fat-Free Mass, *FMI* Fat Mass Index, *FFMI* Fat-Free Mass Index, *ASM* Appendicular Skeletal Muscle, *ASMI* Appendicular Skeletal Muscle Index, *LM* Lean Mass, *DLM* Dry Lean Mass, *LMI* Lean Mass Index, *TAAT* Total Abdominal Adipose Tissue, *ABCD* Amsterdam Born Children and their Development, *ACT* the Adequate Calcium Today project, *ALSPAC* Avon Longitudinal Study of Parents and Children, *Bt20* Birth to Twenty, *FLAME* The Family Lifestyle, Activity, Movement and Eating study, *HELENA* the Healthy Lifestyle in Europe by Nutrition in Adolescence, *HELENA-CSS* the Healthy Lifestyle in Europe by Nutrition in Adolescence cross-sectional study, *IPEN* the International Physical Activity and the Environment Network, *ISCOLE* the International Study of Childhood Obesity, Lifestyle and Environment, *KHANES* The Korea National Health and Nutrition Examination Survey, *MCS* Millennium Cohort Study, *NHANES* National Health and Nutrition Examination Survey, *SWEDES* The Stockholm Weight Development Study, *TRACK* the Transitions and Activity Changes in Kids study.

^a^Indicates abstract only.

^b^Papers have created a categorical or dichotomous variable (i.e underfat/normal fat/excess fat) based on the indicated continuous measured.

^c^Sweden, Austria, Hungary, Greece, Spain, Belgium.

### Characteristics of included studies

There were 38 distinct samples studied across the 50 papers. The Avon Longitudinal Study of Parents and Children (ALSPAC) and a sample from Merida, Mexico were the most commonly included studies, appearing in four and three separate papers, respectively. The majority of papers were conducted in population samples from high-income countries (*n* = 36, 72%) with the remaining papers from MICs (*n* = 14). The UK and the US contributed the most papers (*n* = 10 in the UK, *n* = 8 in the US), with 7 unique studies in both. Sample size across the papers ranged from 74 to 14,314, with a median sample size between 485 and 502.

There was substantial variation in body composition measures used, the definitions of which are outlined in Supplementary Table 1. In this review, we use fat-free measures as a general term referring to any measure of body composition not including fat mass. These measures include FFM, which represents total mass with fat mass excluded, and lean body mass (LBM), a measure of FFM plus essential fats, which are most commonly measured by BIA [78]. Other measures are dry lean mass (DLM), which is LBM without body water, and lean mass (LM), a measure of FFM that excludes bone and is more similar to what is colloquially understood as muscle [79] and is most often measured by DXA. In this review, appendicular skeletal muscle is considered a total body fat-free measure, as muscle mass in the limbs captures 75% of total skeletal muscle mass (SMM), and therefore is a good indicator of total body muscle [79, 80].

Fat measures were considerably more frequently reported than FFM measures (in 46 papers compared to 22), with percentage body fat being the most commonly analysed (29 papers). Among papers that investigate fat-free measures, LM was the most frequently used (8 papers). The majority of papers used either dual x-ray absorptiometry (DXA) (*n* = 25), or bioelectrical impedance (BIA) analysis (*n* = 22) to measure body composition, with two papers using both methods. Five studies used air-displacement plethysmograph with one of these also using BIA. One other paper used deuterium dilution in combination with DXA and plethysmography. The SEP variable most frequently reported was parental education (*n* = 25).

A similar number of studies were conducted in children aged, or with a mean age, between four and ten (*n* = 21) as were conducted in those children and adolescents over the age of ten (*n* = 24). One study was conducted in new-borns and was the only study to be conducted in children under the age of four.

### Childhood SEP and total fat mass measures

Table 3 provides a summary of the patterns of association reported for total FM measures. There were 124 associations tested across 46 papers. Negative associations, where more advantage SEP was associated with lower fat, were reported most often, in 42% (52 association across 31 papers) of the 124 associations. The remaining associations were split between positive associations (27%, 33 associations from 7 papers), where more advantaged SEP was associated with greater fat, and no association (31%, 39 association from 19 papers).

**Table 3:** Summary of associations between socioeconomic position and fat measures in children.

SEP indicator	Direction of SEP and body composition association									Total	
	Positive association			Negative association			No clear/strong direction				
	*N*	%	References	*N*	%	References	*N*	%	References	*N*	%
*Body Fat Percentage*											
Parental Education	6	26%	[77]^b,c^ [77]^a,c^ [74]^c^ [59]^c^ [69]^b,c^ [69]^a,c^	9	43%	[32]^a^ [33]^a^ [43]^a^ [42]^a,c^ [47]^b^ [47]^a^ [46, 58, 73]	8	30%	[32]^b^ [33]^b^ [43]^b^ [42]^b,c^ [60]^c^ [44]^a^ [44]^b^ [52]	23	19%
Composite SEP	4	33%	[69]^b,c^ [69]^a,c^[77]^b,c^ [77]^a,c^	3	25%	[41]^b,c^ [57, 76]	5	41%	[42]^c^ [40]^c^ [37]^a,c^ [37]^b,c^ [72]	12	10%
Occupational Social Class	1	20%	[74]^c^	3	60%	[32]^a^ [42]^c^ [48]	1	20%	[32]^b^	5	4%
Income	1	16%	[74]^c^	2	33%	[71]^a^[71]^b^	3	50%	[60]^c^ [33]^a^ [33]^b^	6	5%
Area or school level SEP^5^	0	–	–	3	75%	[45, 62, 68]	1	25%	[59]^c^	4	3%
PIR	0	–	^–^	3	100%	[65]^a^[65]^b^ [66]	0	–	–	3	2%
School Type	0	–	–	2	66%	[42]^a,c^ [60]^c^	1	33%	[42]^b,c^	3	2%
Miscellaneous	1	25%	[74]^c^	1	25%	[70]^a^	2	50%	[60]^c^ [40]^c^	4	3%
*Body Fat (kg)*											
Parental Education	5	25%	[77]^b,c^ [77]^a,c^ [69]^b,c^ [69]^a,c^ [50]^c^	11	55%	[47]^b^ [47]^a^ [33]^a^ [18]^a^ [32]^a^ [53]^a^ [53]^b^ [54]^a^ [46, 58, 67]	4	20%	[33]^b^ [32]^b^[54]^b^ [34]	20	16%
Composite SEP	5	83%	[77]^b,c^ [77]^a,c^ [69]^b,c^ [69]^a,c^ [56]^a,c^	1	17%	[41]^b,c^	0	–	–	6	5%
Income	0	–	–	2	40%	[29]^b^[29]^a^	3	60%	[33]^b^ [33]^a^ [34]	5	4%
Occupational Social Class	0	–	–	3	75%	[32]^a^ [48, 64]	1	25%	[32]^b^	4	3%
Miscellaneous	1	33%	[50]^c^	0	–	–	2	66%	[50]^c^ [50]^c^	3	2%
*Fat Mass Index*											
Parental Education	4	40%	[77]^b,c^ [77]^a,c^ [69]^b,c^ [69]^a,c^	4	40%	[44]^a^ [36, 55, 73]	2	40%	[44]^b^ [31]	10	8%
Composite SEP	5	63%	[77]^b,c^ [77]^a,c^ [69]^b,c^ [69]^a,c^ [51]^c^	1	12.5%	[75]	2	25%	[30]^a,c^[30]^b,c^	8	7%
PIR	0	–	–	3	100%	[65]^b^ [65]^a^[66]	0	–	–	3	2%
Occupational Social Class	0	–	–	1	33%	[38]^b^	2	66%	[38]^a^ [31]	3	2%
Miscellaneous	0	–	–	0	–	–	1	50%	[36, 39]	2	2%
Overall Distribution of Associations											
*Fat Measures Combined*											
Combined SEP	27%			42%			31%			124	100%
Combined SEP (HIC)	0%			66%			34%			70	56%
Combined SEP (MIC)	61%			11%			28%			54	44%

Positive associations indicate an increase in fat measure with an increase in socioeconomic advantage; inverse associations indicate a decrease in fat measure with an increase in socioeconomic advantage. Miscellaneous SEP measures are where less than two papers reported on the measure.

^a^Indicates results for girls only.

^b^Indicates results for boys only.

^c^Indicates study conducted in a MIC.

In HICs, associations were predominantly negative (66%, 46 associations from 28 papers) with greater socioeconomic advantage being associated with less fat. The remaining associations in HICs all showed no overall pattern of association (24 association from 13 papers). In MICs, the majority of associations were positive (61%) with greater socioeconomic advantage associated with higher levels of fat. Only 11% (6 associations from 3 papers) reported negative associations, with the remaining associations (28%, 15 associations from 6 papers) reporting no overall pattern.

The total body fat measure most frequently reported was FM%, being used 60 times (across 29 papers), followed by FM used 38 times (18 papers), and FMI used 26 times (13 papers). In HICs, using FM or FM% yielded a slightly greater number of negative associations, where greater advantage is related to lower levels of fat, (67% and 68%, respectively) compared to FMI (60%). In MICs, FM and FMI presented almost exclusively positive associations, where greater advantage is related to higher levels of fat (79% and 82%, respectively). FM% exhibited more mixed results with 45% finding positive associations, 17% finding negative and the remaining 38% finding no association.

Parental education was the most commonly used SEP measure across the papers, used in 53 associations across 24 papers. In MICs, composite measures of SEP were the most frequently recorded SEP measure, used in 22 associations across nine papers. Among HICs, negative associations were reported in the majority of associations (≥ 60%) for all SEP measures, with the exception of parental or household income, used in six papers, where no association was most frequently reported (6 association from four papers, out of 10 associations). In MICs, parental education, used in seven papers, yielded a higher number of positive associations (15 association from five papers, out of 18 associations, 83%) compared with composite SEP measures (14 associations from four papers, out of 22 associations, 64%).

**Fig. 2:**
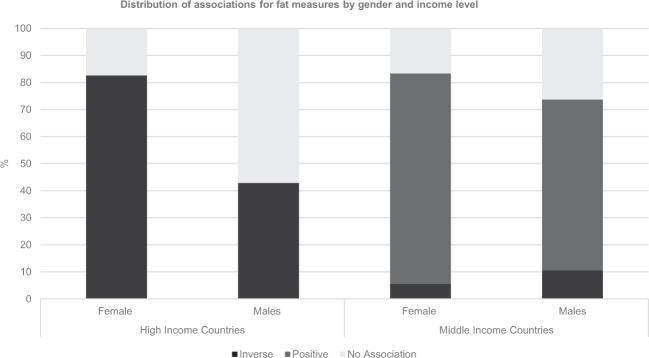
Distribution of associations for fat measures by gender and income level. Girls in HICs (*N* = 23): 83% negative associations, 0% positive associations, 0% non-linear associations, 17% show no association; Boys in HICs (*N* = 21): 43% negative associations, 0% positive associations, 0% non-linear associations, 57% show no association; Girls in MICs (*N* = 18) 6% negative associations, 78% positive associations, 0% non-linear associations, 17% show no association; Boys in MICs (*N* = 9) 11% negative associations, 63% positive associations, 0% non-linear associations, 26% show no association.

Sex-specific analysis was presented in 15 papers in HICs and eight papers in MICs, with 44 and 37 associations reported, respectively (Fig. 2). Negative associations were more frequently reported among girls (83%) compared with boys (43%) in HICs. In MICs positive associations were somewhat more commonly reported in girls (78%) compared to boys (63%). Boys in both HICs and MICs were more likely to show no association between SEP and fat measures (HIC: 57%; MIC: 26%) compared to girls in either (HIC: 19%; MIC: 17%).

When considering results in high quality papers only (rated ≥7* in the quality assessment), findings were similar to the full analysis. In HICs nine associations out of 13 (69%), reported across six papers, showed greater socioeconomic advantage associated with higher levels of fat. In MICs, three out of five associations (60%), reported in two papers, found greater advantage associated with higher FM. In low-quality papers (≤3*) eight out of 13 associations (62%) from five papers reported negative associations similar to that in high quality papers. However, in MICs, the low-quality papers, contrasting with results in the full analysis, showed predominantly negative associations. However, the findings were from only two papers both conducted in the same population sample.

### Childhood SEP and total fat-free mass measures

Table 4 provides a summary of the patterns of association for total body FFM measure. There were 69 associations tested across 22 papers. Approximately half (33 associations in 13 papers) found positive associations (48%), with greater socioeconomic advantage being related to greater FFM. Only 12% demonstrated negative associations, with the remaining 41% reporting no association. Positive associations were reported more frequently in HICs (55%) compared to MICs (43%), whilst negative associations were only reported in MICs (20%).

**Table 4:** Summary of associations between socioeconomic position and fat-free measures in children.

SEP indicator	Direction of SEP and body composition association									Total	
	Positive association			Negative association			No clear direction				
	*N*	%	References	*N*	%	References	*N*	%	References	*N*	%
*Fat-Free* *Mass*											
Miscellaneous	7	100%	[69]^b,c^ [69]^a,c^ [69]^b,c^ [69]^a,c^ [61]^b^ [61]^a^ [48]	0	–	–	0	–	–	7	10%
*Fat-Free Mass %*											
SEP	1	25%	[41]^b,c^	2	50%	[69]^b,c^ [69]^a,c^	1	25%	[42]^c^	4	6%
Parental Education	1	25%	[42]^a,c^	2	50%	[69]^b,c^ [69]^a,c^	1	25%	[42]^b,c^	4	6%
Miscellaneous	3	75%	[61]^b^ [61]^a^ [42]^a,c^	0	–	–	1	25%	[42]^b,c^	4	6%
*Fat-Free Mass Index*											
Occupational Social Class	1	33%	[38]^a^	0	–	–	2	66%	[38]^b^ [31]	3	4%
Parental Education	0	–	–	0	–	–	3	100%	[73]^d^ [31, 36]	3	4%
Miscellaneous	1	50%	[36]	0	–	–	1	50%	[75]	2	3%
*Dry Lean Mass*											
SEP	3	100%	[42]^c^ [41]^b,c^ [40]^c^	0	–		0	–	–	3	4%
Miscellaneous	2	40%	[42]^b,c^[42]^b,c^	0	–		3	60%	[42]^a,c^ [42]^a,c^ [40]^c^	5	7%
*Lean Mass*											
Parental Education^1^	3	43%	[77]^a,c^[77]^b,c^[58]	0	–	–	4	57%	[18]^a^ [50]^c^[32, 49]	7	10%
SEP	3	100%	[77]^a,c^[77]^b,c^[56]^a,c^	0	–	–	0	–	–	3	4%
Occupational Social Class	0	–	–	0	–	–	3	100%	[32, 49, 64]	3	4%
Miscellaneous	0	–	–	0	–	–	4	100%	[50]^c^[50]^c^[50]^c^[49]	4	6%
*Lean Mass %*											
Parental Education	1	33%	[58]	2	66%	[77]^a,c^[77]^b,c^	0	–	–	3	4%
SEP	0	–	–	2	100%	[77]^a,c^[77]^b,c^	0	–	–	2	3%
*Lean Mass Index*											
SEP	0	–	–	0	–	–	3	100%	[51]^c^[77]^a,c^ [77]^b,c^	3	4%
Parental Education	0	–	–	0	–	–	2	100%	[77]^a,c^[77]^b,c^	2	3%
*Appendicular Skeletal Muscle*											
School Level SEP	2	100%	[61]^a^[61]^b^	0	–	–	0	–	–	2	3%
*Appendicular Skeletal Muscle %*											
Miscellaneous	5	100%	[63]^a^ [63]^b^ [61]^a^ [61]^b^ [52]	0	–	–	0	–	–	5	7%
Overall Weighted Distribution of Associations											
*Lean Body Measures* *Combined*											
Combined SEP	48%			12%			41%			69	100%
Combined SEP (HIC)	55%			0%			45%			29	42%
Combined SEP (MIC)	43%			20%			38%			40	58%

Positive associations indicate an increase in fat measure with an increase in socioeconomic advantage; inverse associations indicate a decrease in fat measure with an increase in socioeconomic advantage. Miscellaneous SEP measures are where less than two papers reported on the measure.

^a^Indicates results for girls only.

^b^Indicates results for boys only.

^c^Indicates study conducted in a MIC.

^d^Indicates lean body mass instead of FFM.

Raw fat-free measures, used in 14 papers, show positive associations in 59% of analyses (20 associations coming from nine papers, out of 34 associations), more frequently than both percentage measures (50%, 11 associations coming from six papers, out of 22 associations across eight papers) and considerably more often than indexed measures (15%, two associations coming from two papers, out of 13 associations across five papers). Measures that include bone in their assessment show positive associations slightly more often (54%) than those which exclude bone (42%).

Parental education was the most frequently investigated SEP measure, used in 24 associations. Composite measures of SEP were also frequently used (*n* = 18), with all except one such association tested in MICs. Parental occupational social class and measures of area-level SEP were used in eight and nine associations respectively, although for area-level SEP, eight were all tested in the same paper. Parental income was used three times in two papers. In MICs there was a slightly higher number of positive associations reported when using composite measures of SEP (53%) compared to education (40%). In HICs, approximately one-third of associations with both education and occupational social class were observed to be positive. In the small number of analyses including area-level SEP and income in HICs, only positive associations were seen.

**Fig. 3:**
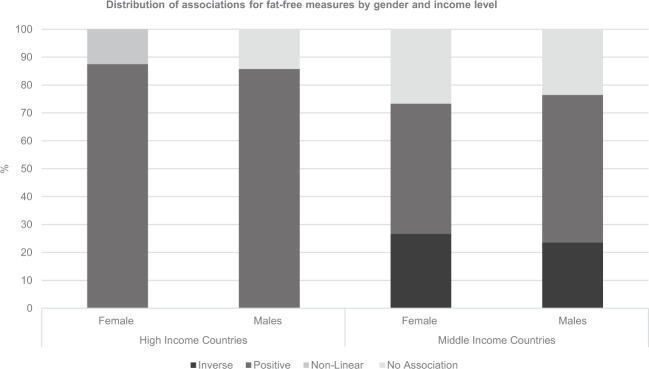
Distribution of associations for fat-free measures by gender and income level. Girls in HICs (*N* = 8): 0% negative associations, 87.5% positive associations, 12.5% non-linear associations, 40% show no association; Boys in HICs (*N* = 7): 0% negative associations, 86% positive associations, 0% non-linear associations, 14% show no association; Girls in MICs (*N* = 15) 27% negative associations, 47% positive associations, 0% non-linear associations, 27% show no association; Boys in MICs (*N* = 17) 24% negative associations, 53% positive associations, 0% non-linear associations, 24% show no association.

In both HICs and MICs, only five papers presented sex-specific analysis, with 15 and 31 associations reported, respectively. Positive associations were reported 86% of the time in boys, and 88% of the time in girls (Fig. 3). Results for sex-specific analysis in MICs was more similar to the pooled results, although there were slightly greater number of positive associations in boys (53%) compared with girls (46%).

In high quality papers (≥7*) the percentage of positive associations reported were lower than in the full analysis, with only three out of 12 associations (25%), from seven papers, finding greater socioeconomic advantage related to higher FFM, and the rest reporting no association. Similar to the full analysis, HICs reported positive associations more frequently with three out of seven associations (43%), from five papers, whilst in MICs all five associations coming from only two papers reported no overall pattern. In low quality papers (≤3*), the number of positive associations in HICs is lower compared to the full analysis with three of nine associations (33%) from four papers reporting such a finding. In MICs, the seven positive associations out of 12 (58%) were from just two papers, both using the same sample population.

### Childhood SEP and ratio and distribution measures

Five papers reported on the association between SEP and a ratio or distribution measure (Table 5). Two papers used the same sample from the US and the remaining papers reported results from the UK (*n* = 2) and Brazil (*n* = 1).

**Table 5:** Results of associations between socioeconomic position and childhood ratio and distribution measures.

Paper	Country	*N*	Age	Study or description of the population	SEP measure	Body composition measure	Findings
Willig et al. [75]	USA	254	7–12	Children from ongoing cross-sectional study, whose parents classified them through self-report as either African American, European American, or Hispanic American.	SEP	Trunk FMI	SEP was negatively associated with trunk FMI (all at *p* < 0.05). Increase in advantage was associated with decreases trunk FMI.
Cardel et al. [35]	USA	267	7–12	Children self-identifying as African American, European American, or Hispanic American from Birmingham, Alabama area.	SEP	Trunk FM, TAAT	Increase in social advantage associated with decreases in central adiposity.
Ness et al. [64]	UK	5 917	10	ALSPAC	Social Occupational Class	Trunk FM	No association
Magalhaes et al. [59]	Brazil	183	4–7	Children aged 4–7 from a retrospective cohort who were monitored for the first months of life by a support program to breastfeeding (PROLAC) in the city of Vicosa, southeast Brazil.	Mothers education, Income per capita	%Android Fat	No association
McCarthy et al. [61]	UK	2 297	5–14	Caucasian children from inner city London and from more affluent surrounding counties.	School Level SEP	Muscle:Fat Ratio	Lower muscle to fat ratio in low-income groups in all age groups, except for females aged 11–14.

*SEP* Socioeconomic Position, *FMI* Fat Mass Index, *TAAT* Total abdominal adipose tissue, *ALSPAC* Avon longitudinal Study of Parents and Children.

Four papers looked at the association between SEP and a measure of central fat. Two of these using the same sample found greater social advantage to be associated with decreases in trunk FM, total abdominal adipose tissue [35] and trunk FMI [75]. The other two papers found no association between any SEP variable considered and central fat [59, 64]. Only one paper looked at a ratio measure and found a lower mean muscle to fat ratio in lower parental income groups, except in girls aged 11–14 [61].

## Discussion

This systematic review finds evidence of socioeconomic inequalities in body composition in childhood and adolescence, although the direction and strength of these inequalities varies by measure of SEP, measure of body composition, sex and economic development of the country of study. Evidence of negative associations were generally observed for all measures of fat. Negative associations were more frequently observed in samples from HICs compared with MICs, with positive associations only observed in studies from MICs. In HICs, negative associations were found more frequently in girls compared with boys, whilst in MICs girls more often showed positive associations. Greater socioeconomic advantage was associated with greater FFM in approximately half of the associations studied, but such associations were less common with outcome measures indexed to body size in both HICs and MICs, and in studies rated as higher quality. The review highlighted a lack of research using area-level measures of SEP, parental income and using more detailed measures of body composition, such as ratio and distribution measures.

Our findings for fat mass are broadly consistent with those from reviews using BMI as the measure of adiposity. Shrewsbury and Wardle [2] and Barriuso et al. [3], found associations between greater socioeconomic disadvantage and higher levels of adiposity among children and adolescents from HICs, identifying almost no associations in the opposite direction. Sobal and Stunkard [4] and Dinsa et al. [81] observed consistent evidence of more disadvantaged SEP being related to lower levels of obesity among children in MICs. However, where Sobal and Stunkard [4] found the association between SEP and obesity, measured largely by BMI, to be inconsistent among children in HICs, we found more consistent evidence that greater socioeconomic disadvantage was associated with higher fat mass.

Previous research using anthropometric measures of obesity have reported that, in HICs, studies using parental education report inverse associations most frequently [2, 3] consistent with our findings. Area-level measures of SEP have previously been shown to be particularly strong predictors of obesity [26], and this may be due to the close link between area-level measures of SEP and obesogenic elements in the environment [82, 83]. However, we found very few studies using area-level SEP and body composition.

There are a greater percentage of negative associations between SEP and fat measures among children compared with those found in our review of adults in HICs [20]. This difference may indicate life course differences in the association between SEP and adiposity, or secular changes in inequalities given that the studies conducted in children typically include individuals born more recently than those conducted in adults. A comparison of the British birth cohorts demonstrated increasing inequalities in BMI with age within the cohorts, and inequalities in childhood and adolescents were only observed in the most recently born cohorts [84]. Research using the Fels Longitudinal Study demonstrated a secular increase in FM% in children and adolescents from 1960 to 1999 [13]. Our results on directly measured adiposity in children compared to our results in adults [20] broadly match the trends seen in studies demonstrating secular increases in the inequalities in BMI [15, 84, 85]. Follow up of childhood cohorts into adulthood will be needed to distinguish a secular trend from an age effect.

Most studies in this review were conducted in children born post 1984, which means that in HICs they were all born into an obesogenic environment, the onset of which is generally estimated to be in the 1980s [86, 87]. Disadvantaged SEP, after the onset of the obesogenic environment, has been associated with increased proximity to fast food outlets [88, 89], larger advertising of fast food [90], and worse access to sports facilities [91] and green spaces [92] in HICs. Children are particularly influenced by advertising [93] and the food environment [94] and are less likely than adults to have a beneficial relationship between the built environment and levels of physical activity [95].

In contrast to HICs, disadvantaged SEP was associated with lower levels of body fat for children in MICs. Children of advantaged SEP in these countries have greater exposure to a western lifestyle compared to those of disadvantaged SEP, and in particular greater access to more expensive and energy dense foods [81]. Transnational food companies that have expanded to MICs often target children with their adverts, therefore making children particularly vulnerable to their efforts to increase purchase and consumption in MICs [96]. In addition, differences in physical activity may play a role. A study from India found higher rates of obesity in private schools compared with government schools [97], explained in part by a greater reliance on cars or buses to get to private schools, whilst children who attended government schools were more likely to walk or cycle [96]. There may also be cultural differences in perception of obesity between HICs and MICs [98], with overweight children in MICs being considered healthier by parents [97] and poorer understanding of the health consequences of obesity among mothers in MICs [99].

We previously reported on the association between SEP and fat-free measures in adulthood, finding predominantly no association, although with slight evidence of positive associations among women in HICs [20]. In contrast, we found considerable evidence for inequalities in fat-free measures in childhood, especially in HICs, and with few differences in associations between boys and girls.

Greater inequalities in FFM in childhood compared to adulthood may reflect a secular decline in levels of FFM, which are likely to be accompanied by growing inequalities, in the opposite direction to inequalities seen for adiposity. The Fels Longitudinal Study has shown mean FFMI to be lower in boys born in the 1990s compared with boys of the same age born decades before [13]. Serial data more recently has shown a secular decline in muscle strength, measured by handgrip, sit-ups, bent-arm hang and standing broad-jump tests, among children in the UK [14]. It is likely that secular changes in body composition would coincide with secular change in the inequalities, as has been observed with BMI [84], especially as changes to body composition have occurred alongside an overall increase in health inequalities [100]. Peak muscle function is determined across childhood and early adulthood and then maintained through midlife [101], and early development has been shown to be an important determinant of LM in later adult life [102]. It is therefore probable that inequalities in FFM observed in more recent generations in children are likely to persist into adulthood and old age.

In our review, few studies used indexed measures of FFM that aim to, at least partially, remove the correlation with height, nor did they adjust for height. Among those that did, there were fewer observed positive associations. Associations of greater disadvantage and lower FFM may therefore be explained, at least partially by height, as there is evidence that disadvantaged SEP is associated with shorter height across childhood and adulthood in most populations [103], although in HICs there is evidence this inequality has narrowed [84]. Positive associations in FFM in MICs may, however, also reflect that adequate nutrition is required for the development of muscle tissue as well as height, specifically intake of protein and micronutrients [104–108]. Increases in fat mass are accompanied by adaptive increases in lean mass [17, 109, 110] and this may explain the association between disadvantaged SEP and lower fat-free measures in MICs, as children in disadvantaged SEP are more likely to be food insecure and lack essential macro and micronutrients [111], and therefore be shorter and have lower levels of both FM and FFM [112]. Further studies which appropriately adjust FFM measures for height are required to assess this. Few studies adjusted for fat mass, which is a suggested way of identifying the independent inequalities in lean mass.

### Strengths and limitations

This review was registered with PROSPERO and has been carried out according to the published protocol [21]. The review has a generous inclusion criterion, capturing a broad range of evidence, thereby reducing selection bias. We also reduced bias by having two independent reviewers conducting each stage of the review, including selection of studies into the review and extraction of data, as well as completion of a quality assessment which was used to inform of the variability in study quality.

The generous inclusion criteria resulted in considerable heterogeneity in samples, study design and measures used. This variation, together with heterogeneity in the analytical approaches and reporting of results, prevented us from being able to conduct a meta-analysis. Additionally, the association, not paper, was used as the unit of analysis since most papers reported more than one association, meaning that in some cases one paper may contribute more weight to the overall summary of findings. The same data sets were also used by multiple authors in multiple papers.

As it was not possible to conduct a meta-analysis, it was not possible to assess publication bias. It is, however, possible that publication bias exists with papers based on small sample sizes showing positive results are more likely to be published than those showing null findings. We did, however, include studies that tested the association of interest as part of a wider set of analyses instead of just focussing on those studies with a specific hypothesis on SEP differences, which may have reduced the impact of publication bias. This included papers that did not report full results where associations were found to be non-significant in preliminary analysis. However, this does mean that associations may not have appropriate adjustment for confounders.

This review prioritised assigning patterns to associations using the effect estimates and confidence intervals, which convey more about the direction and strength of effect, and the accuracy of these estimates [113, 114] and to overcome problems related to a reliance on *P* values [114, 115]. However, many of the papers included reported *P* values alongside only descriptive data or description of the association in the text. As *P* values are influenced by the sample size of the study, lack of associations observed in such studies is likely due to a lack of statistical power. The studies included in this review were generally small, with a median sample size between 485 and 502. Additionally, because of the heterogeneity in SEP measures, outcomes, statistical approaches and the reporting of results it was not possible to make comparisons of effect size across papers, even among those studies that did use appropriate statistical methods. There may also have been overadjustment as studies have adjusted for factors which may actually be mediators rather than confounders.

There is inconsistency in the literature relating to the terminology used to describe FFM [116]. It is not uncommon for the same terminology to be used for different measures of FFM, or indeed different terminology to be used for a single measure. We sought to ensure comparability of results by applying standard definitions of FFM measures across the review, but some papers did not provide enough clarity on the measures used to do this confidently. There is a need for consistent definitions to be applied across the body composition literature, and for authors to provide clarity on the measures used.

We amended protocol slightly, due to the need to analyse heterogeneity by country income level, as more papers were identified from MICs than expected and it was clear that this was an unignorable source of heterogeneity. We had no papers included from LICs, limiting the ability to explore SEP and body composition associations in countries at an earlier stage of the nutrition transition, which would have been valuable for understanding the changing relationship of SEP and obesity with economic development.

### Implications

In monitoring inequalities, BMI may accurately capture fat mass at a population level in childhood, given the similarities of our findings with reviews on social inequalities in BMI. However, our review suggests contrasting findings on inequalities in FFM in HICs. This may, assuming such associations are not fully explained by inequalities in height, mean that BMI underestimates the inequalities in the health risks related to adiposity. If children from more disadvantaged SEP groups have lower levels of muscle mass and strength, as well as higher levels of fat mass, this may have important implications for inequalities in outcomes which also require good muscle function. As we have found greater evidence of inequalities in body composition in children compared with adults, tracking of body composition through the life course in more recent generations could have important implications for inequalities in physical capability in later life. Follow up of these childhood cohorts is needed to confirm whether these are secular rather than age-related changes in inequalities.

It is also crucial that future research appropriately indexes body composition measures and distinguishes measures that include bone from those that do not. Inequalities in height are likely to be an important factor that can explain the observed inequalities in such measures. The results of this review also highlight the need for more research investigating the associations of SEP with ratio and distribution measures of body composition which are related to metabolic and cardiovascular disease outcomes, especially in MICs, and greater research on sex differences in both HICs and MICs. There are also gaps in research looking at the effect of area-level measures on body composition, a measure of SEP that is closely linked to the obesogenic environment, particularly in relation to FFM.

Efforts should be made to address inequalities in both FM and FFM among children in HICs and MICs by reducing access to, and advertising of, fast food to children, and promoting and ensuring equal access to healthy and nutritious food. Promotion of physical activity and access to sport facilities should also be prioritised in poorer communities to address inequalities in FFM.

## Supplementary information

Supplementary File 1 - PRISMA Checklist

Supplementary File 2 - Altered Newcastle-Ottawa Quality Assessment Form

Supplementary Table 1
