# Supplementary material for: Socioeconomic position and body composition in childhood in high- and middle-income countries: a systematic review and narrative synthesis
Source: Int J Obes (Lond). 2021 Jul 27;45(11):2316–34. doi: 10.1038/s41366-021-00899-y (PMC8528703; doi:10.1038/s41366-021-00899-y)
Supplement: Supplementary file 2 — Altered Newcastle-Ottawa Quality Assessment Form [file 41366_2021_899_MOESM2_ESM.docx]

## Supplementary File 2. Quality Assessment Form

**NEWCASTLE - OTTAWA QUALITY ASSESSMENT SCALE (amended)**

Note: A study can be awarded a maximum of one star for each numbered item within the Selection and Outcome categories. A maximum of two stars can be given for Comparability

**Selection**

1) Representativeness of the exposed cohort/study

a) Truly representative of the source population **🟑**

b) Somewhat representative of the source population**🟑**

c) Selected group of users e.g. nurses, volunteers

d) No description of the derivation of the cohort

2) Ascertainment of SEP

a) Prospectively from parents/family/own (or linking to area-level indicators) **🟑**

b) Structured interview (recall) **🟑**

c) Written self-report

d) No description

**Comparability**

1) Comparability of cohorts on the basis of the design or analysis

a) Study controls for birth weight **🟑**

b) Study controls for any additional relevant factors (e.g. age, sex, ethnicity) **🟑**

c) Only unadjusted model presented

**Outcome**

1) Assessment of body composition

a) Measure indexed to body size or ratio (i.e fat mass index or fat:lean ratio) **🟑**

b) No indexation or ratio

c) No description

2) Was follow-up long enough for outcomes to occur (if *a)* go to 3a, if *b)* go to 3bi and 3bii)

a) Longitudinal **🟑**

b) Cross-sectional

3a) For longitudinal -Adequacy of follow up of cohorts

a) Complete follow up - all subjects accounted for **🟑**

b) Subjects lost to follow up unlikely to introduce bias - small number lost - > 75% follow up, or description provided of those lost) **🟑**

c) Follow up rate < 75% and no description of those lost

d) No statement

3bi) For Cross sectional - Sample size:

a) Justified and satisfactory. **🟑**

b) Not justified.

3bii) For Cross sectional - Non-respondents:

a) Comparability between respondents and non-respondents characteristics is established, and the response rate is satisfactory. **🟑**

b) The response rate is unsatisfactory, or the comparability between respondents and non-respondents is unsatisfactory.

c) No description of the response rate or the characteristics of the responders and the non-responders.

4) Statistical test:

a) The statistical test used to analyze the data is clearly described and appropriate, and the measurement of the association is presented, including confidence intervals and the probability level (p value). **🟑**

b) The statistical test is not appropriate, not described or incomplete.

≥ 7 **🟑** = high quality

< 7 **🟑** = low quality
